# Implementation of pharmacist-led intervention on asymptomatic bacteriuria treatment in adults patients

**DOI:** 10.1017/ash.2026.10841

**Published:** 2026-09-09

**Authors:** Gabriela Maria Urreta, Alice Margulis Landayan, Jeyma Fernandez, Jorge Murillo, Stephen Breazeale, Timothy Gauthier

**Affiliations:** 1 South Miami Hospital, USA; 2 Baptist Health South Florida Inc, USA

## Introduction

Asymptomatic bacteriuria (ASB) is the presence of bacteria in the urine without symptoms of infection (ie, dysuria, urgency, frequency, suprapubic pain).^1^ The incidence of ASB is around 45% in women and 50% in individuals residing in long-term care facilities.^2^ Current evidence-based guidelines recommend against antimicrobial treatment in most patients, as ASB is generally benign. Exceptions include pregnant individuals, individuals undergoing endourologic procedures associated with mucosal trauma, and those within one month of renal transplantation.^3^

Treatment of ASB has not been shown to improve outcomes and is associated with harms, including increased risk of *Clostridioides difficile* infection, antimicrobial resistance, and prolonged hospital length of stay.^4^ Despite these risks, inappropriate antibiotic use remains prevalent, with up to 75% of patients with ASB receiving unnecessary treatment.^5^

Several antimicrobial stewardship studies have supported provider education and decision-making guidance to decrease inappropriate treatment of ASB, while also highlighting the important role of pharmacists in optimizing antimicrobial appropriateness and treatment duration.^6–8^ The aim for this quality improvement project was to assess the impact of pharmacist-led interventions on decreasing the use of antimicrobial therapy for ASB.

## Methods

This was a single-center retrospective evaluation conducted at a 450-bed community teaching hospital. Adult patients admitted for ≥24 hours and initiated on antimicrobials for ASB were included. Patients with indications for ASB treatment (ie, pregnancy, undergoing endourologic procedures with mucosal trauma, or recent history [<1 month] of renal transplant), chronic urinary tract infection (UTI) antimicrobial prophylaxis, fever (≥38 °C), sepsis, receiving Infectious Diseases-restricted antimicrobials, infections requiring antimicrobial therapy, one-time antimicrobial doses, incarcerated individuals, comfort measures only, or hospice were excluded.

A pharmacist-led ASB intervention was implemented January 1^st^, 2026 through March 31^st^, 2026, consisting of pharmacist education, a standardized ASB assessment algorithm (Supplementary Figure 1), and a daily antimicrobial review report enabling real-time interventions. Pharmacists utilized the algorithm to differentiate ASB from symptomatic UTI and identify opportunities for antimicrobial discontinuation or duration optimization. Recommendations were communicated directly to prescribers through either verbal discussion or electronic secure messaging software. During weekdays, using a Discern Analytics® 2.0 report, designated pharmacists identified active antimicrobial orders with selected indications for “UTI,” “cystitis,” or “pyelonephritis” and assessed for appropriateness. Providers were contacted with intervention recommendations where appropriate.

Outcomes from the postimplementation period were compared to a preimplementation period in which no standardized ASB algorithm or dedicated review process existed (January 1^st^, 2025 through March 31^st^, 2025). The primary outcome was duration of antimicrobial therapy in days. Secondary outcomes included proportion of patients who received at least one antimicrobial-related pharmacist intervention, number of antimicrobial-related pharmacist interventions, adverse drug events, hospital length of stay, and 30-day infection-related outcomes. A sample size of 102 patients (51 per group) was estimated to detect a 50% reduction in therapy duration with 80% power. Categorical data was analyzed using *χ* ^2^ test or Fisher’s exact test and continuous data was analyzed with Mann-Whitney U test. Statistical significance determined by *P*-value *< .05*. This project was deemed exempt from Institutional Review Board review.

## Results

A total of 291 patients preimplementation and 221 patients postimplementation were screened, with 60 and 40 patients included, respectively. The most common reason for exclusion was presence of urinary symptoms. Baseline and microbiological characteristics are presented in Table 1. Median duration of antimicrobial therapy decreased from 7 days preimplementation to 2 days postimplementation (*P < .*001).

**Table 1.** Baseline characteristics and microbiological findings Table 1 long description.

	Pre-implementation (*n* = 60)	Post-implementation (*n* = 40)	*p*-value
Median age (IQR) – years	81 (70-87)	82 (73-86)	.79
Female sex, *n* (%)	41 (68)	24 (60)	.39
Race or ethnicity, *n* (%)			
Hispanic/Latino	34 (57)	22 (55)	.87
White/non-Hispanic	23 (38)	15 (38)	.93
Black/African American	3 (5)	3 (7)	.61
Altered mental status, *n* (%)	11 (18)	10 (25)	.42
History of dementia, *n* (%)	11 (18)	4 (10)	.22
Urinary catheter, *n* (%)	11 (18)	8 (20)	.66
Leukocytosis, *n* (%)	12 (20)	11 (28)	.38
Urine culture obtained, *n* (%)	54 (90)	30 (75)	.31
*Urine culture reflexed from urinalysis*	46 (77)	27 (67)	.05
*Urine culture ordered without reflex*	8 (13)	3 (8)	.74
Urine culture pathogen, *n* (%)			
*Escherichia coli*	16 (30)	6 (20)	.34
*Klebsiella pneumoniae*	6 (11)	6 (20)	.33
*Proteus mirabilis*	5 (9)	1 (3)	.41
*Enterococcus faecalis*	5 (9)	3 (10)	1.00
*Pseudomonas aeruginosa*	4 (7)	6 (20)	.16
*Candida spp.*	3 (6)	2 (7)	1.00
*Staphylococcus epidermidis*	0 (0)	1 (3)	.36
*Streptococcus agalactiae*	0 (0)	1 (3)	.36
*No growth*	15 (28)	4 (14)	.13
Urine culture with ESBL producing pathogen, *n* (%)	5 (10)	2 (7)	1.00

IQR: interquartile range; ESBL: extended-spectrum beta-lactamase.

Successful pharmacist interventions occurred in 7 patients (12%) preimplementation compared with 36 patients (90%) postimplementation (*P < .*0001). Total pharmacist interventions increased from 7 to 37 following implementation, most notably driven by antibiotic discontinuation (2 vs 24, *P < .*0001) and shortened treatment duration (4 vs 12, *P = .*004). No antimicrobial-related adverse drug events were identified. Median hospital length of stay was longer in the postimplementation group (3 vs 5 d; *P = .02*); however, no 30-day infection-related deaths or UTI-related readmissions occurred in either group. Overall, 90% of postimplementation pharmacist interventions were accepted. Altered mental status was the most common reason for intervention rejection.

## Discussion

Implementation of a pharmacist-led ASB intervention was associated with significant increases in pharmacist interventions and reductions in antimicrobial therapy duration, without increased 30-day infection-related mortality or UTI-related readmissions. These findings support guideline recommendations against the treatment of ASB in most patient populations due to the lack of clinical benefit from and the potential harm associated with unnecessary antimicrobial exposure.^3^ Limiting antimicrobial exposure and decreasing duration may reduce the risk of adverse drug events, *C. difficile* infection, and antimicrobial resistance.^3^

Our findings are consistent with prior studies demonstrating that pharmacist-led stewardship interventions can significantly reduce inappropriate antibiotic use in ASB.^9^ The high rate of accepted postimplementation pharmacist interventions highlights the feasibility and effectiveness of integrating pharmacists into routine stewardship workflows.

Despite these improvements, provider discomfort with discontinuing antimicrobial therapy was observed, particularly among patients with dementia or altered mental status who may be unable to reliably report urinary symptoms. Although these patients were assessed using the same standardized ASB criteria, diagnostic uncertainty remained a common barrier to intervention acceptance. Diagnostic uncertainty in such populations is a well-recognized contributor to inappropriate ASB treatment and remains a significant barrier, highlighting the need for further targeted education and clinical decision support tools that reinforce evidence-based diagnostic criteria.^4^

Our study has several limitations. The retrospective single-center design and small sample size may limit generalizability. The short study period limits assessment of long-term sustainability. The increased length of stay observed postimplementation likely reflects non-infectious factors, as no differences in infection-related outcomes were identified. The smaller sample size in the postimplementation group compared to the preimplementation group suggests that more patients with ASB in the postimplementation period were not prescribed antimicrobial therapy, likely secondary to ongoing discussions with providers, which contributed to increased education and appropriate ASB management.

Overall, pharmacist-led interventions targeting ASB represent a valuable component of ASP programs. By reducing unnecessary antimicrobial exposure without compromising outcomes, such interventions support high-quality evidence-based care and advance institutional stewardship goals.

## Supporting information

10.1017/ash.2026.10841.sm001 Urreta et al. supplementary material Urreta et al. supplementary material

## Supplementary material

The supplementary material for this article can be found at https://doi.org/10.1017/ash.2026.10841.

## Table 1. Long description

A table comparing baseline characteristics and microbiological findings pre-implementation and post-implementation. The table has 18 rows and 5 columns. The columns are labeled Pre-implementation (n = 60), Post-implementation (n = 40), and p-value. The rows include various characteristics and findings such as Median age (IQR) - years, Female sex, n (%), Race or ethnicity, n (%), Altered mental status, n (%), History of dementia, n (%), Urinary catheter, n (%), Leukocytosis, n (%), Urine culture obtained, n (%), Urine culture reflexed from urinalysis, Urine culture ordered without reflex, Urine culture pathogen, n (%), and Urine culture with ESBL producing pathogen, n (%). Each row provides specific data points for pre-implementation and post-implementation groups, along with corresponding p-values. Notable trends include the percentage of urine culture obtained decreasing from 90 percent pre-implementation to 75 percent post-implementation, and the median duration of antimicrobial therapy decreasing from 7 days preimplementation to 2 days postimplementation.

Navigate back to Table 1..
