# Supplementary material for: Implementation of pharmacist-led intervention on asymptomatic bacteriuria treatment in adults patients
Source: Antimicrob Steward Healthc Epidemiol. 2026 Sep 9;6(1):e265. doi: 10.1017/ash.2026.10841 (PMC13760413; doi:10.1017/ash.2026.10841)
Supplement: Supplementary file 1 [file S2732494X26108419sup001.docx]

**Supplementary Material**

Supplementary Figure 1. Asymptomatic bacteriuria algorithm

Supplementary Table 1. Antimicrobial Treatment

|  | **Pre-Implementation**  **(n = 60)** | **Post-Implementation**  **(n = 40)** | **P-Value** |
| --- | --- | --- | --- |
| Initial antibiotic, n (%)  *Ceftriaxone*  *Piperacillin/tazobactam*  *Cefepime*  *Other* | 51 (85)  4 (6)  2 (4)  3 (5) | 34 (85)  0 (0)  0 (0)  6 (15) | 1.00  0.15  0.52  0.15 |
| Initial antibiotic route of administration, n (%)  *Intravenous*  *Oral* | 58 (97)   2 (3) | 35 (88)   5 (12) | 0.11  0.11 |
| Initial ordering provider specialty, n (%)  *Internal Medicine*  *Emergency Medicine*  *Cardiology*  *Critical Care*  *Nephrology*  *Infectious Diseases* | 30 (50)  28 (46)  1 (2)  0 (0)  0 (0)  1 (2) | 25 (63)   5 (12)   4 (10)   4 (10)  2 (5)  0 (0) | 0.22  **<0.0001**  0.15  **0.02**  0.16  1.00 |
